# Attentional Effects on Phenomenological Appearance: How They Change with Task Instructions and Measurement Methods

**DOI:** 10.1371/journal.pone.0152353

**Published:** 2016-03-29

**Authors:** Britt Anderson

**Affiliations:** Dept. of Psychology/Centre for Theoretical Neuroscience, University of Waterloo, Waterloo, ON, Canada; University of Verona, ITALY

## Abstract

It has been reported that exogenous cues accentuate contrast appearance. The empirical finding is controversial because non-veridical perception challenges the idea that attention prioritizes processing resources to make perception better, and because philosophers have used the finding to challenge representational accounts of mental experience. The present experiments confirm that when evaluated with *comparison* paradigms exogenous cues increase the apparent contrast. In addition, contrast appearance was also changed by simply changing the purpose of a secondary task. When comparison and discrimination reports were combined in a single experiment there was a behavioral disassociation: contrast enhanced for comparison responses, but did not change for discrimination judgments, even when participants made both types of judgment for a single stimulus. That a single object can have multiple simultaneous appearances leads inescapably to the conclusion that our unitary mental experience is illusory.

## Introduction

For over a century psychologists have been interested in how attention may change appearance as consciously perceived [1]. And for the last decade this topic has been reanimated by the ingenious experimental procedure of Carrasco and colleagues as employed for a number of sensory dimensions in e.g., [2–6]. Applied to contrast, the key procedural innovation was to have participants *compare* which of two candidate stimuli had the greater contrast, and then, as a secondary task, *discriminate* the stimulus orientation. One of the stimuli was of a fixed, standard contrast. The other stimulus, the test, varied in contrast and orientation. The investigators inferred stimulus contrast on the basis of psychometric functions. These functions were fit to the probability that participants used the hand on the side of the test stimulus. The resulting claim, that “attention alters appearance”, has been a controversial one.

An early objection was that the pattern of choice probability used to infer attentional effects on contrast appearance could have been due to response biases. In their original report Carrasco, Ling and Read [6] performed two types of control experiments. First, the time between the presentation of the cue and the stimuli was increased. Since the window for attentional effects is constrained [7] this should abolish the effect if it was attentional in nature, and it did. Second, participants reported which of two stimuli had *lower* contrast. Despite this change in instructions, Carrasco, Ling and Read [6] reported a similar shift in the point of subjective equality. Subsequently, much of the discussion has been about whether difference judgments or equality judgments are the better procedure, and what the proper statistical procedures are (see for example: [8–14]).

In addition to the technical critiques, there has been a second variety of objection that focuses on conceptual issues. A common framework for interpreting attentional effects is that of prioritization. We are, so the argument runs, overwhelmed with sensory data, and we cannot process it all [15]. The role of attention is to prioritize what is important [16, 17] so that the important input can receive preferential treatment. As a result, attended stimuli should be perceived more accurately, should look more like what is “out there” [18–21]. Carrasco, Ling and Read [6] is a challenge to this logic, because Carrasco, Ling and Read [6] found not only that attention *altered* appearance, but that it *exaggerated* it.

A third point of interest for these data is their philosophical implications. In *Mental Paint*, Block [22] relies extensively on these results to argue that direct realist and representationalist accounts of mental experience are necessarily incomplete. Beck [23] and Schneider [24] respond to these arguments with salience based accounts. Stazicker [25] focuses on variable precision.

In summary, the claim that attention alters phenomenal appearance is a contested one with important implications for psychology, neuroscience, and philosophy of mind. The experiments reported here set out to explore whether particular aspects of the nature of the psycho-physical judgment could influence the shift in apparent contrast. The principal findings are that the intention of a judgment alters appearance, and that despite our seamless experience a single stimulus may have multiple appearances.

## Methods

All experiments were variations of the same basic method. The details of the procedures and participants are consolidated here.

### Stimuli Presentation

Testing was performed at a desk with a computer in a quiet room. Participants sat approximately 60 cm from the CRT display.

Stimuli were presented on a conventional CRT monitor (1040 × 768 pixels, 85 Hz refresh, Width 33 cm) controlled by a personal computer. The experimental program was written in Python and made use of the Psychopy Python library [26]. The monitor’s contrast was linearized with the use of a ColorCAL Colorimeter (Cambridge Research Systems, Rochester, UK).

**Fig 1:**
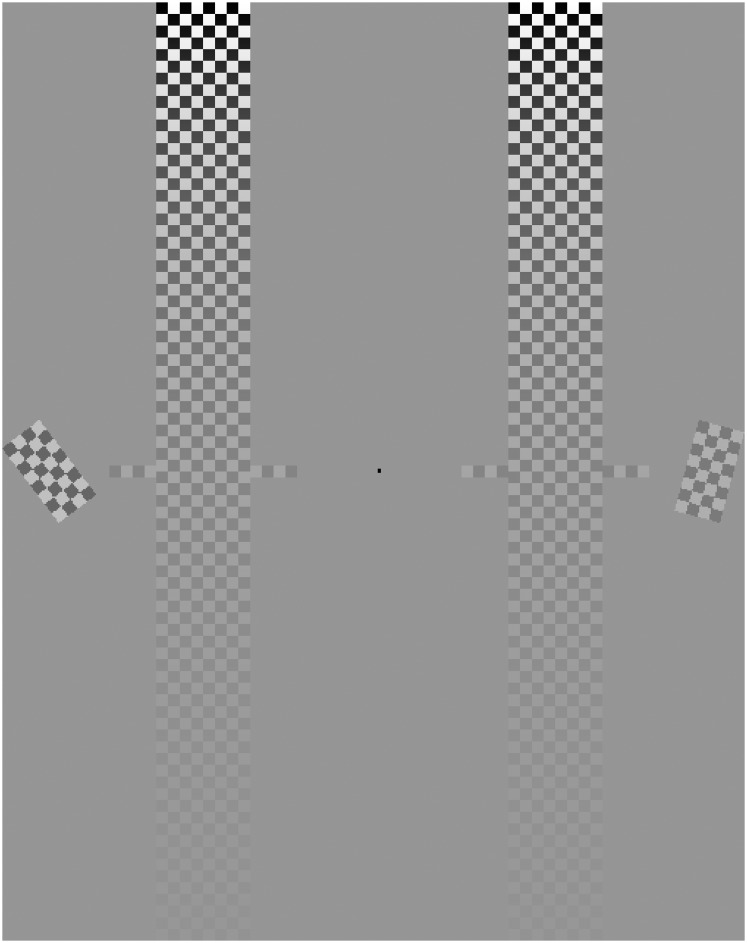
Screenshot of Test Stimuli and Report Meter. Following a brief interval, oriented “checkerboards” appeared on the left and/or right (depending on the experiment). Participants were instructed when reporting orientation to rotate the response meter on the appropriate side. For example, in Experiment 1 the appropriate side would have been the left as that is the side with the checkerboard of higher contrast) to match the orientation of the checkerboard. For Experiment 4 the appropriate side would have been the right as that checkerboard is tilted more to the right. When participants were to report contrast the small “wings” on either side of the response meter could be slid up and down to indicate the level of the contrast of the checkerboard. Different keys controlled the rotation and contrast reports (it was only possible to make the variety of response each Experiment required). A different key “locked-in” the participant’s choice and then the next trial began.

Experimental trials began with a fixation dot presented centrally (see Fig 1). In addition, there were two long, vertically oriented meters to the right and left of fixation. The meters were 19 degrees tall, and 1.9 degrees wide. The distance from the fixation spot to their midpoint was 3.5 degrees. Their texture was black and white checked and followed a log contrast ramp from top (1.00) to bottom (0.02). The top of the response meter had a luminance of 66.1 cd/m^2^. The luminance of the background gray was similar at 71.3 cd/m^2^. On the same monitor white measured 116 and black <1 cd/m^2^.

After a delay of 500 ms an exogenous, luminance cue was presented. It was 0.3 degrees in diameter, and appeared either 7 degrees to the right, 7 degrees to the left, or centrally. When the cue appeared to the right or left it was immediately above the checkerboard stimuli. It was presented 2.5 degrees above the level of the fix spot, which made it about 0.5 degrees above the uppermost edge of the stimuli (this varied slightly with the tilt of the checkerboard stimuli). Neutral cues were presented at the center of the screen (overwriting the fixation spot). The cue remained on screen for 71 ms.

After 59 ms, two checkerboard stimuli were shown for 47 ms. The checkerboard stimuli were located 7 degrees of visual angle to the left and right of the fixation point. These stimuli were both 1.9 degrees by 0.9 degrees, and were also composed of black and white squares. The reason for not going with a grating type stimulus is that with Gabor type gratings changing the contrast can change the apparent size of the stimulus (low contrast gratings may look smaller). Also, the contrast is not uniform across the grating. The contrast of one of these small checkerboard stimuli, the *standard stimulus*, was always 0.22 while the contrast of the other, the *test stimulus*, was selected uniformly from the range 0.06 to 0.79. Thus, there were not “levels” of contrast as in [6]. The checkerboard stimuli were also both rotated. The *standard stimulus* was always oriented 15 degrees clockwise, while the orientation of the *test stimulus* was selected uniformly from between −45 degrees and +45 degrees.

After the stimuli disappeared, the participants responded. They either rotated the tall, checked rectangle to match the orientation of the small checkerboard stimulus that had had the greater contrast, or adjusted the level of two “wings” to match the contrast shown. The response method varied across experiments and participants were only exposed to one response method. Participants were instructed in advance, and had practice trials to confirm their understanding. A right set of buttons controlled the right meter and a left set of buttons controlled the left meter. Participants adjusted the response meter to their satisfaction, and then “locked it in” with a different button press. The trial was then concluded and the next trial immediately followed.

There were 10 practice trials, and five blocks of 50 test trials. For the practice trials, the timing was slowed down to better enable demonstrating the task. In addition, there was visual feedback. After each trial, a new response meter was projected on to the display. It revealed the correct response (orientation or contrast). It was shown on the side of the correct response. In addition, there were two auditory tones that played after each trial. If the participant made his response using the meter on the correct side and within a predefined tolerance they received a “bing” feedback tone. Otherwise they received a “donk” feedback tone. Participants were not given details about the magnitude of the error that provoked the two different tones, but they were informed that the “bing” was more accurate than the “donk.” The purpose of the tonal feedback was to help participants maintain their motivation and alertness over the course of the experimental session. In prior tasks of this sort, I found better participant engagement and tolerance with this vague form of feedback than none at all.

### Statistics and Curve Fitting

All statistical tests used the R statistical language [27]. For comparing the effects of cuing as a factor mixed models were used with participants as a random factor [28]. These analyses used the lme function of the nlme package [29]. Comparison across factors used the glht function from the multcomp package [30]. For comparing model fits, the Akaike information criterion was used [31]. Comparisons of proportions used a logistic regression. This procedure looks at the probability of events as a function of possible factors. Significance tests used Chi square statistic and were implemented using the glm, and anova functions.

Two different psychometric curve fitting procedures were used: conventional, and expanded. The conventional Weibull fitting procedure was intended to match the curve fits in [6]. Weibull fits were implemented with the R statistical language [27], used the glm function, log transformed contrast values, the binomial family, and the cloglog link function. For fitting psychometric functions with possibly different parameters for location, slope, and asymptote the R functions provided by Lindsey at http://www.commanster.eu/rcode.html, were used as described in [32, 33]. Curves were also fit to binned and unbinned data with no significant changes in fits. This was done because in Carrasco, Ling and Read [6] the test stimulus contrast was binned, while in the current experiments the contrast and orientation of the stimulus was drawn from a continuous distribution. For Weibull fits, data were partitioned into 15 subsets of roughly equal size (the number 15 was used to be comparable to the numbers of bins used in Carrasco, Ling and Read [6]).

The scripts used for the statistical analyses can be found with the data at https://osf.io/tnp9h/.

### Experiment 1 Participants

Participants signed an informed consent before proceeding. Twenty participants were enrolled in the protocol and nineteen completed it (six men; average age = 20). They were all undergraduates at the University of Waterloo receiving course credit in exchange for their research participation. The protocol had the approval of the University of Waterloo Office of Research Ethics. Task duration, including signing the informed consent, instructions, testing, and debriefing took a little less than an hour. Note that no participant participated in more than one experiment in this series.

### Experiment 2 Participants

Twenty different undergraduate participants took part in this experiment. The methods were identical to those of Experiment 1 except only the test stimulus was drawn on the screen. This kept all aspects of the experiment the same as Experiment 1. Participants were instructed to reproduce the orientation of the displayed checkerboard. They were not instructed to compare the two possible stimulus locations. It is important to note that the only real difference between Experiments 2 and 1 is that the “standard” stimulus of Experiment 2 had a contrast of 0.

### Experiment 3 Participants

Twenty-one additional undergraduate participants took part in Experiment 3, and twenty completed all trials. The apparatus and protocol were identical to Experiment 2 except that participants were instructed to report the contrast of the stimulus (rather than, as in Experiment 2, the orientation). The range of contrasts and orientations of stimuli was identical to Experiment 2 and to the test stimuli of Experiment 1 as the same computer code was used to generate the stimuli for all three experiments.

For the same reasons as outlined in Experiment 2, the two cues that occurred at locations on the screen where there was no stimulus were coded as “invalid” cues, and the cues that were proximate to stimulus locations were coded as “valid.”

### Experiment 4 Participants

Twenty participants took part in this experiment. The procedures and stimuli were identical to Experiment 1 with the following exception: Instead of being instructed to select based on contrast and to report orientation, they were instead instructed to select based on orientation and to report contrast. The standard stimulus was oriented 15 degrees clockwise of vertical (as it had been in Experiment 1 as well; it’s contrast was also fixed at 0.22—as it had been in Experiment 1) and participants were told to respond on the side with the more right-tilted checkerboard. Their response was to report the contrast of this checkerboard stimulus by adjusting the height of “wings” along a varying contrast meter, as is shown in Fig 1, to the height of the row that best matched the checkerboard’s contrast.

### Experiment 5 Participants

Twenty two undergraduate participants were enrolled and twenty completed the protocol. The same stimuli were used as in Experiment 1. Participants were instructed to report the contrast of the stimulus that had the greater contrast and to use the response keys on the same side as the stimulus that they were reporting.

## Results

### Exogenous Cues and Apparent Contrast: Paired Stimuli—Comparison Paradigm

The first experiment reported here is essentially a replication of Carrasco, Ling and Read [6]. Critically, it uses two stimuli on every trial, a test and a standard, and infers effects on contrast from psychometric functions fit to comparison data, that is the probability that participants choose the test stimulus. In brief, two small “checkerboard”-like stimuli were presented. One stimulus, the “standard”, always had a contrast of 0.22 and was rotated 15 degrees clockwise. The other stimulus, the “test”, varied in contrast and orientation. Participants rotated a response meter to the orientation of the checkerboard with more contrast by using the hand on that side (see Fig 1 for a screenshot of the basic task display). Cues were small black dots that appeared either at the center of the screen (neutral; 20% of the trials), or above the stimulus with the higher/lower contrast with equal probability and were thus uninformative.

**Fig 2:**
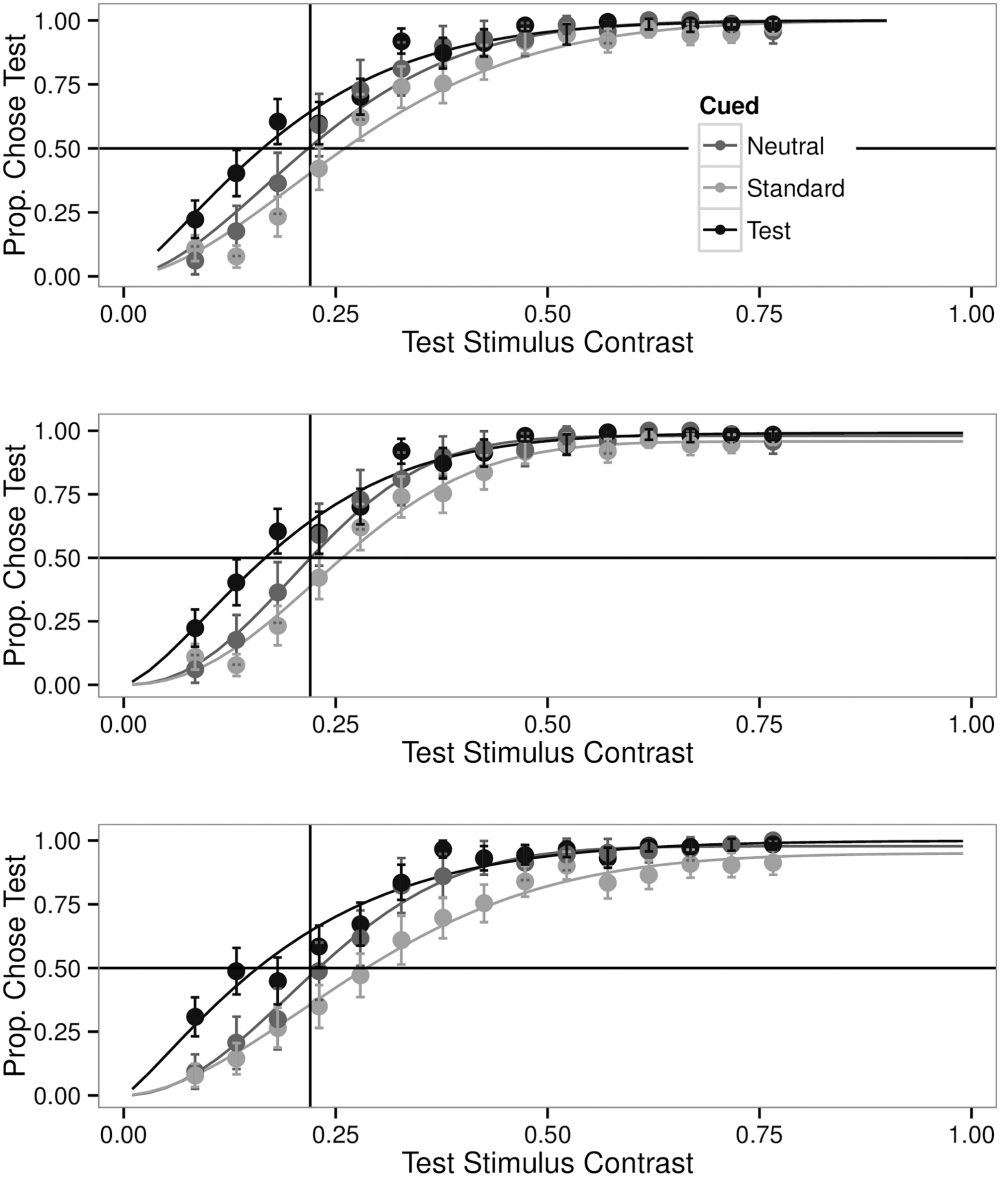
Performance Data and Weibull Fits. Top: Trials from Experiment 1 were divided into 15 equal sized bins based on the contrast of the test stimulus. For each participant, the proportion of trials for which they chose the test stimulus as the more contrasted was computed for each of the three cue conditions. The points show the mean and one standard error. The lines show standard psychometric functions fit to a standard Weibull function using the glm of R. This is similar to the method reported in Carrasco, Ling and Read [6]. Middle: Same data fit to a Weibull function that contains cue specific terms for the slope, location, and upper asymptote. This results in a significant improvement in fit over the lines shown in the Top panel (see Table 1 for AIC values). Bottom: The data from Experiment 5 are fit with the same methodology as the Middle panel, and show the same pattern of results.

The data from this first experiment (Fig 2) replicate the pattern found in Carrasco, Ling and Read [6]. When participants detect which of two stimuli were of greater contrast, and use this information to make an orientation report, Weibull functions fit to the probability of choosing the test stimulus show a leftward shift in the point of subjective equality when they are preceded by a luminance cue, and a rightward shift when the cue is on the opposite side. As the test stimulus contrast was a continuous variable, linear mixed models can be used to test the effects of contrast and cuing and are provided below. But to provide comparability, and as test stimuli were binned in [6], the test stimuli contrast from this experiment were also subdivided into 15 equal sized bins post hoc and these binned data analyzed by ANOVA (though it is notable that no ANOVAs were presented in the original report [6]). The main effect of contrast (F(14) = 167.34), the cue factor (F(2) = 28.55), and the interaction (F(28) = 2.77) were all statistically significant (all p’s < 0.001).

However, the empirical choice probabilities do not asymptote at 1 and it has been demonstrated [34] that small differences in the upper asymptote of empirical choice functions may have large effects on psychometric curve fitting. Therefore, the analysis was repeated using a series of functions that permitted fitting for differences in slope, asymptote, and location written by Lindsey, and described in [32].

Fig 2 (Middle) shows the psychometric functions when the Weibull fit is allowed to have separate slopes, locations, and upper asymptotes. Table 1 shows the AICs for several combinations of function parameters. The model which includes components for the different cue conditions for each of slope, location, and upper asymptote, has the best explanatory power [35]. This best model (Fig 2 Middle) reveals that the trials where the standard stimulus is cued have a finite probability to be chosen even when the test stimulus has maximal contrast. And at the lower end of the scale, test cued stimuli also have a non-zero choice probability when they are at the minimum contrast.

**Table 1:** AICs for Psychometric Fits.

Factors	*AIC*
Contrast only	3481.56
Contrast, Slope	3475.3
Contrast, Location	3388.86
Contrast, Upper Asym	3447.09
Contrast, Upper Asym, Location	3388.86
Contrast, Upper Asym, Slope	3429.66
Contrast, Slope, Location	3639.81
Contrast, Upper Asym, Slope, Location	3363.22

Another observation from the fully fitted model is that the *neutral* cued trials have a steeper slope than the other two cued conditions. This can be seen in the way the neutral cued trials are close to the standard cued trials for low test contrast, and rapidly meet the test cued trials after the test stimulus exceeds the standard contrast. This brings the neutrally cued trials into closer alignment with the optimally performing function, which is a step function that transitions from 0 choice probability below 0.22 to 1.0 for a test stimulus greater than 0.22.

This observation, that neutral cuing results in a psychometric function closest to optimal, is confirmed by accuracy. Recall that the participants are instructed to select the stimulus with the higher contrast. A correct response is when they in fact do this. We can compare accuracy as a function of cue condition by comparing the proportion of correct trials thus defined. The probability correct for each of the participants in each cue condition was computed and compared with a linear mixed model where participant identity was a random factor and cue condition was the fixed factor. Compared to the neutral condition, on standard cued trials participants were correct 3.7% less often (t = −2.5, p = 0.016) and for the test cued trials they were correct 4.6% less often (t = −3.1, p = 0.0034). The test and standard cued conditions did not significantly differ from each other (p = 0.81, Tukey’s Post Hoc Test). Numerically, none of the participants had their most accurate peformance for the test condition.

These data indicate that the effects of uninformative luminance cues on choice probability are more complex than appearance shifts alone, and that response bias probably plays a role. This can be inferred from the less than perfect performance at the extremes of test stimulus contrast. Even at maximal contrast (0.79) the test stimulus is not always chosen if the standard has been cued. At the other extreme, at minimal contrast (0.06), the test stimulus will occasionally be chosen as having the greater contrast if it has been preceded by a cue. This leads one to suspect that just as in Prinzmetal, Long and Leonhardt [12] participants might pick empty space as having greater contrast were that empty space cued—a clear indicator of some contribution of response bias to choice behavior.

The psychometric functions also show differences in the point of subjective equality. When the test stimulus has been preceded by an uninformative luminance cue a contrast of less than the standard is needed for the test and standard stimuli to be chosen as the more contrasted equally often. The situation is reversed when the standard has been cued. As expected, when neither has been cued, the neutral condition, the point where they are equally likely to be chosen as having the greater contrast is when they in fact are of equal contrast.

An additional interesting finding is that the “best” performance does not come when one of the stimuli has been cued, but in fact when neither has. The slope is steepest for the neutral condition, and this is also the cue condition with the best behavioral performance. As the task is to compare two stimuli, and the neutral cue is the only condition that does not bias the participant to one location or the other, this result make sense. It also emphasizes the interplay between task and attentional effects.

In summary, this first experiment replicated the results of Carrasco, Ling and Read [6]. When a participant chooses between two visible stimuli, and the effect on contrast is inferred from comparison performance, then there is an increase in apparent contrast.

### Exogenous Cues Produce Response Bias—Orientation Reports

The data from Experiment 1 reveal that there is a shift in the point of subjective equality as a function of which of two stimuli are cued. That pattern is consistent with either a change in appearance or response bias. The second experiment examined the issue of response bias in greater detail. With two stimuli on every trial, as there were in the first experiment, it is not possible to disambiguate errors from contrast shifts. If a participant makes their report on the side of the stimulus with the objectively lower contrast is that because of phenomenological contrast enhancement, a mistaken button push, or a response bias? If there is only one stimulus on the screen, then the first possibility can be eliminated.

Therefore, the second experiment was a repeat of the first experiment *except the contrast of the standard was 0.00*. This meant that there would only be one stimulus drawn on the screen for every trial. In addition, the two cue conditions in the first experiment that were labeled neutral and standard were now labeled invalid, as they both cued a spatial location where no stimulus was present (and for consistency the trials when the test stimulus position was cued were labeled valid).

**Fig 3:**
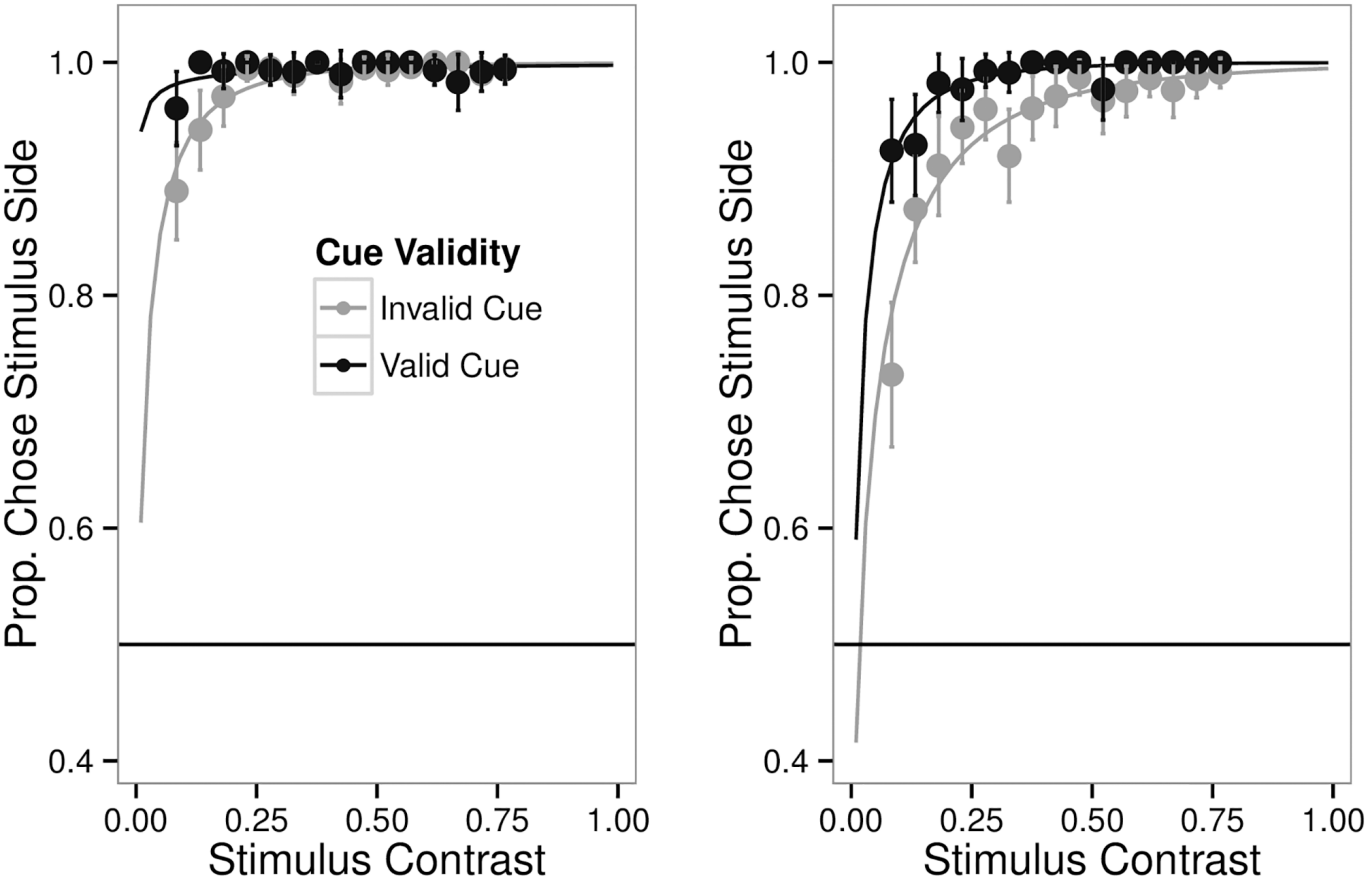
Probability of Making a Response on the Side Where the Stimulus Was Shown as a Function of Cue Validity. Left. Instructions were to report the stimulus’s orientation (Experiment 2). Right. Instructions were to report the stimulus’s contrast (Experiment 3). While the data of Experiments 2 and 3 are qualitatively similar, participants were more likely to make errors when the instructions required them to focus on contrast as opposed to orientation even though the range of contrasts and orientations presented was the same for both experiments.

Fig 3 (Left) shows the choice probability for validly and invalidly cued trials. People make mistakes. They do try to report the orientation of empty space. And they are more likely to try and do this if that location has been preceded by an uninformative luminance cue.

As the task was not a speeded task, the errors are not likely to be anticipations as nothing prevented participants from beginning to respond on one side and then changing to the other. It also seems unlikely that the errors are simply due to participants not being able to see the stimulus as overall performance was high. The difference in accuracy as a function of cue validity is statistically significant (t = 3.6, p = 0.0021, linear mixed effect model).

In addition to demonstrating that response bias pertains in the Carrasco Cuing Paradigm, these data are relevant for noise reduction models of attention [36, 37]. Following the logic of Eckstein, Drescher and Shimozaki [38] we would predict that if attention is working by noise reduction then participants should be better at distinguishing empty space as such when it is cued as there would be less “noise” at that location. However, participants are not less likely to respond on the wrong side when cued there, but rather more so.

If one is seeking to find differences between the first and second experiments as a way of arguing that response bias might pertain in the second, but not the first, then one could highlight that the the contrast judgment in the first experiment was an explicit instruction to use the hand on the side of the stimulus with greater contrast, while in the second experiment it was implicit, use the hand on the same side as the stimulus. To eliminate this concern the second experiment was repeated with the judgment one of contrast: report the contrast with the hand on the same side of the stimulus. Thus the participants were explicitly directed to the importance of contrast in their performance.

### Exogenous Cues Produce Response Bias—Contrast Reports

This third experiment followed the methods of one and two. Again, the standard contrast was 0.0, so only one stimulus, the test, was visible on every trial. As participants had to respond with the hand on the same side as the stimulus there was an implicit selection of the side with greater contrast (something versus nothing), but as the discrimination response required the participants to reproduce the contrast of the stimulus they were explicitly aware that contrast was a pertinent variable.

The results from this experiment were very similar to Experiment 2, but they were not identical. Fig 3 (right panel) shows the choice probability for validly and invalidly cued trials. The mean and standard error of the choice proportions are shown for binned data and are superimposed on Weibull fits. Fig 3 (right panel) again reveals that people make mistakes. Again, they are more likely to report the contrast of an invisible stimulus when that location is cued.

Overall accuracy was high, and was modulated by cue validity. The improvement in accuracy with cuing (defined as choosing the side with the stimulus) was statistically significant (t = 3, p = 0.0073, linear mixed effect model). An interesting, unexpected finding was that the probability of picking the side with the stimulus differed between Experiments 2 and 3. This was despite the fact that all the parameters were the same, and despite the fact that the choosing of the response side was based on identical stimuli. The only difference between the two experiments was what the ultimate nature of the discrimination report would be: orientation or contrast. It is important to note that performance on the discriminating task is not the difference that is being highlighted. What was different was the proportion of times participants correctly identified which side contained the stimulus. A combined linear mixed model analysis with factors for experiment, cue validity, and their interaction revealed a significant effect for cue validity (t = 3.6, p = 0.0017). The experimental factor was also significant (t = 3.5, p = 0.0014). The interaction term was marginal (t = −1.9, p = 0.066).

This third experiment confirmed the contribution of response bias to choice in the Carrasco Cuing Paradigm. In addition, this experiment demonstrated that false localization proportion is susceptible to task instructions. It is not simply an effect of stimulus contrast or the presence of a luminance cue. When participants prioritize contrast information they are paradoxically less accurate at locating low contrast stimuli than when they are prioritizing orientation information. This observation demonstrates that what we see is not merely a consequence of what we are shown, or what we are doing, but also what we are looking for.

### Exogenous Cues and Apparent Contrast: Paired Stimuli—Discrimination Paradigm

In the conventional Carrasco Cuing Task the inference of what a person consciously perceived is based on a parameter derived from fitting a psychometric function to a collection of data recorded over many trials. This is rather indirect, and it does not provide any trial by trial measure of performance for comparison. However, these data can be measured if the basic task is flipped. That is, a participant selects their response side on the basis of orientation, and makes a direct report of the contrast they were shown. Such a manipulation provides an alternative method to assess cue effects on contrast, it also provides a follow-up on how perception may be affected by the nature of a secondary task.

The fourth experiment was a repeat of the original paired stimuli experiment (see page 8), but with the orientation and contrast judgments reversed. Participants made their response using the hand that was on the same side as the stimulus that was more right tilted. They then reported the contrast using the response meters.

For this experiment there was no effect of cue condition (test side, standard side, or neutral) on the probability of choosing the correct side (the one that was tilted more to the right). All p-values were > 0.7. This suggests that an uninformative luminance cue does not bias the appearance of a tilted rectangular checkerboard, even when the tilt of that object is the basis for a challenging selection. It also suggests that response biases are not the complete story for the contrast effect reported by Carrasco, Ling and Read [6]. Of note, Carrasco has also found stimuli for which the cue does not shift judgments, e.g. hue [3]. One suggestion has been that the shift in perceptual intensity is only found for sensory attributes that have a natural sense of “greater” such as luminance or color saturation, but not hue. The argument could be modified to suggest that response and decision biases are only naturally biased when a stimulus dimension has a character that suggests a natural magnitude.

Contradicting the result that luminance cues increase contrast, the direct trial by trial reports of contrast showed no effect of cue status. A linear mixed effects model was fit with the dependent variable of contrast reported and whether the participant was responding to the cued side as the independent variable. The identity of the participant was included as a random factor. The effect was non-significant (F (2, 38) = 1.69, p-value = 0.2). The reported contrast on the cued side was not on average different from the non-cued side.

**Fig 4:**
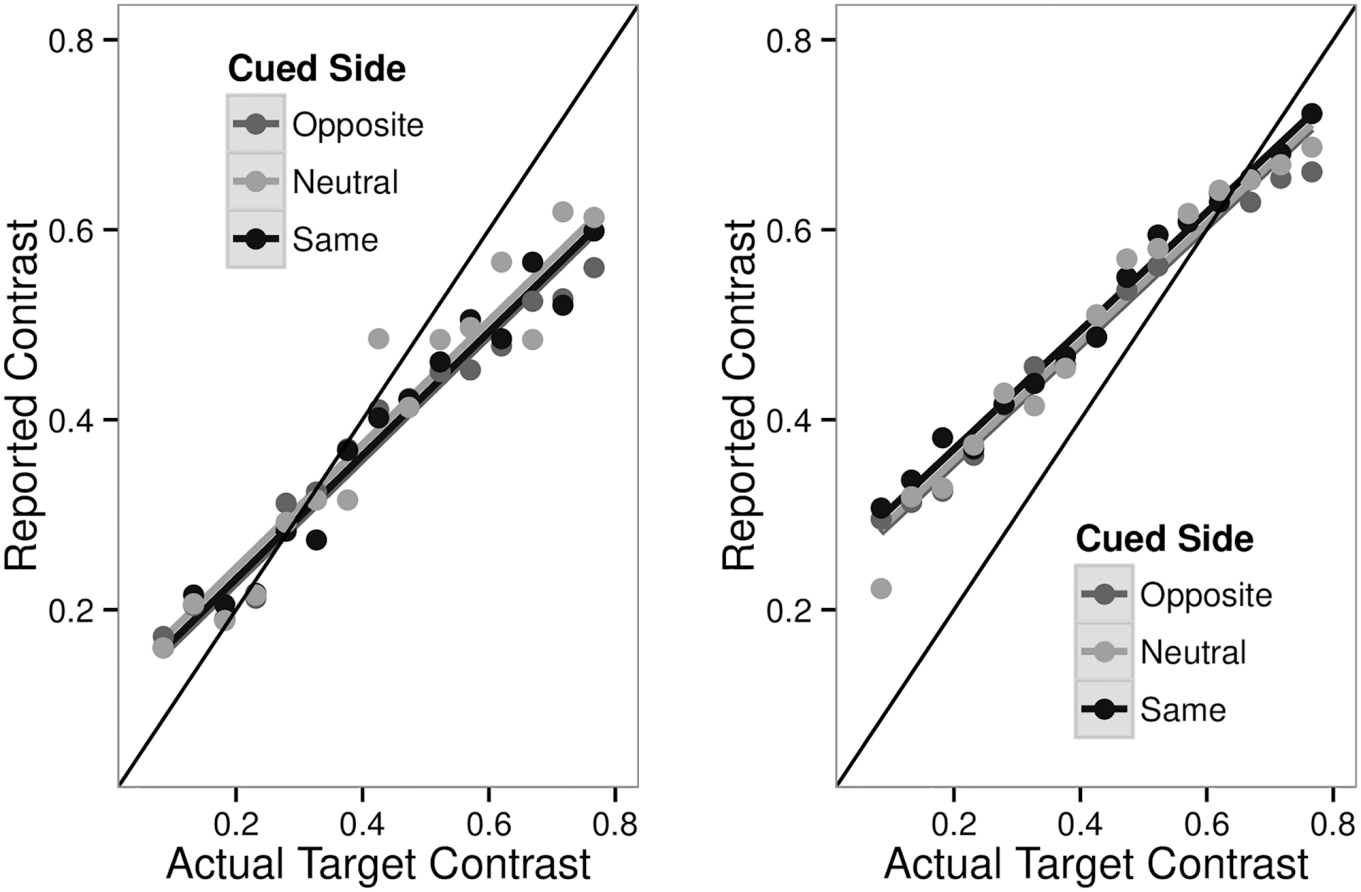
Comparing Contrast Judgements. Left panel: The relationship between reported contrast and contrast shown on that side subdivided by the side of the cue. Binned data are shown as circles and lines show the linear fits. There is no difference for the magnitude estimates as a function of cue status in Experiment 4. Right panel: Same analysis for the data of Experiment 5. There is again a robust correlation between displayed and judged contrast, but no effect of the cue. In both panels the thin black line has slope 1, and represents perfect discrimination. For both data sets typical magnitude judgement effects are found: contraction bias (smaller stimuli are overestimated and larger stimuli are underestimated) and centering bias (estimates center on the modal or most frequent stimuli) [39].

This null result cannot be attributed to generally poor performance. Linear regression models with and without a factor for whether the response side was the cued side (using the glm function in R) confirmed that participants made reliable judgments about contrast, and that there was a significant relation between the contrast shown and contrast reported (t (df = 2) = 61.71, p value essentially zero). More critically, adding the cue factor did not result in any significant improvement in fit (Deviance = 0.05, df = 2, p-value = 0.22; Chi Square). This relation can be visualized by plotting the mean contrast for each participant for representative bins of judged contrast (Fig 4). The participants contrast judgments were not altered based on whether they were judging a cued or uncued stimulus.

In summary, within the same basic experimental paradigm the effect of cuing on contrast appearance varies depending on whether the inference is based on comparisons and psychometric curve fitting, or whether it is based on discrimination and direct contrast reports. Is this discrepancy due to chance, or is it actually related to the nature of the judgment? The finding of this experiment implies that a person could give two different contrast values for the exact same stimulus.

### Exogenous Cues and Apparent Contrast: Paired Stimuli—Comparison Paradigm and Discrimination Paradigm

This last experiment represents a hybrid that demands two separate reports of contrast for each stimulus. First, participants make their report using the hand on the side of the more contrasted stimulus, and second they provide a direct match of the contrast they were shown. From the first set of data we can repeat the analysis used in the first experiment, fitting psychometric functions, to look for a shift in the point of subjective equality. From the second set of data we can repeat the analysis used in the fourth experiment to directly compare displayed and reported contrast.

Fig 2 shows the effects of exogenous cues on choice probabilities for this experiment (bottom panel) and the first experiment (top and middle panels). From this analysis one could conclude that an uninformative luminance cue alters appearance. Fig 4 shows the effects of exogenous cues on contrast reporting for this experiment (right panel) and the fourth experiment (left panel). They are concordant. From this analysis one could conclude that an uninformative luminance cue does not alter appearance.

One way to try and explain away the discrepancy would be to assert that attentional effects on contrast are brief, and decay before the discrimination report. There are three reasons this seems unlikely. First, there is a good agreement between the displayed and the reported contrast. Second, because participants clearly remember the contrast shown. One would have to posit that it is only the “attentional” component of contrast that is forgotten. And third because delayed effects have been reported previously in a similar task looking for cue effects on speed perception [40].

Addressing the second point, there are other attentional effects observable in the later discrimination reports, so one would have to posit that it is only the attentional effect on contrast, and not other attentional effects that are forgotten. For example, the time to initiate a response (RT) and the accuracy for discrimination judgments were measured for all experiments. An omnibus linear mixed effects model for all experiments was conducted for the logarithm of RT (note that participants were instructed to respond as accurately as possible. Speed was not a priority). Trials on which the participants responded to the cued side were significantly faster than invalidly cued trials (t value = −6.3, p value = 0.000) and neutral trials (t value = −7.28, p value = 0.000), which did not differ from each other (t value = 2.03, p value = 0.103).

An omnibus test for the accuracy on orientation judgments was made using the two experiments that required orientation reports (Experiments 1 and 2). Absolute orientation difference was measured between the participant response and the orientation of the stimulus on the side where the participant responded, and only included trials where participants responded on the side of a stimulus. Accuracy for orientation judgments was affected by cuing. Trials on which the participant responded to the cued side had a smaller absolute orientation error than invalidly cued trials (t value = −2.21, p value = 0.0689) and were not significantly different from neutrally cued trials (t value = 0.474, p value = 0.883). Neutrally cued trials were also judged more accurately than invalidly cued trials (t value = −2.23, p value = 0.0662).

An omnibus test for accuracy on contrast judgments was made using the three experiments that required contrast reports (Experiments 3, 4 and 5). Contrast accuracy was computed as the absolute difference in the participant’s contrast report and the contrast of the stimulus on the side of the participant’s response. Accuracy for the contrast judgments also showed cuing effects, but the magnitude of the effects was small. Trials on which the participant responded to the cued side were not judged differently from invalidly cued trials (t value = −0.54, p value = 0.851). Neutrally cued trials were judged more accurately than validly cued trials, but the difference was not significant (t value = 2.02, p value = 0.106). Neutrally cued trials were however judged with a significantly smaller contrast error than invalidly cued trials (t value = −2.41, p value = 0.0413).

For the last point, similar delayed effects of cues have been found by others. Valsecchi, Vescovi and Turatto [40] reported a study of speed estimation using a similar protocol. Two drifting gratings were presented preceded by a small black dot as an exogenous cue. Participants made two judgments. They made a comparison judgment about which of the two drifting gratings was moving faster, and they made an equality judgment. In their Experiment 3 the equality judgment came first, and the comparison came second. There was a 1 second delay between the two reports. Despite this healthy delay, and no differential shift of the speed estimates in response to cuing, the comparison judgment at this delayed interval still showed a shift in the point of subjective equality for the cued side consistent with it being perceived as faster than the uncued side.

In summary, the fact that the discrimination judgment came later than the choice report seems unlikely to account for the failure to see a shift in contrast. Contrast reports at this time were in good agreement with the displayed, actual, contrast. Other measures of attentional effects are present in the discrimination reports. And prior work in other laboratories has shown persistence of cuing on comparative judgments at even longer delays [40].

## Discussion

The results of [6] have been much discussed, and are controversial. Given that the replicability for psychology has recently been estimated to be as low as 30% [41], it is reasonable to ask first how reliable these results are? Shortly after Carrasco, Ling and Read [6] published their study, Schneider [14] reproduced similar differences in choice behavior, but with a protocol that varied in some ways from that of Carrasco, Ling and Read [6], e.g. the effect was found with white cues rather than black cues. Prinzmetal, Long and Leonhardt [12] also reproduced the basic finding, and their Experiment 1 used a protocol very similar to that of Carrasco, Ling and Read [6]. In the present study, Experiment 1 was the experiment that most closely mimicked the procedures of Carrasco, Ling and Read [6], but there were differences. Contrast was chosen from a continuous distribution and was not binned. After choosing the side with greater contrast, participants made a continuous orientation response, and not simply a binary clockwise or anti-clockwise choice. These procedural differences appear not to have been critical though, as the basic change in choice behavior was replicated. As the basic empirical result is robust to procedural changes, and has been replicated in several different laboratories, we can focus on the claim that the basis for the differences in choice behavior mean that attention accentuates contrast. The results reported here are neither a confirmation nor contradiction of that result (or perhaps they are both). The results do call in to question the presumption that attentional consequences are *uniquely* determined by the combination of a cue and the sensory input.

The results reported here suggest that response bias contributes to performance in a Carrasco Cuing Task, but it is probably not a complete explanation. The data in favor of response bias playing a role is the result in two separate experiments that participants pick cued, empty space as having greater contrast. As there is no contrast to be enhanced, response bias seems the simplest explanation. On the other hand, in the experiment when participants were instructed to choose the more right tilted of two stimuli, there was no shift in their choice probability as a function of cuing. It can’t therefore be that cues simply function as a “thumb on the scale” when choosing between two similar alternatives.

Judging contrast by a 2AFC task is not a simple matter. The choice made reflects multiple processes. First, is the perceptual component. Second, comes a decision where the agent decides which of the two stimuli was of greater contrast. Third, there is the selection of the response. It is not necessary that the response selected be completely determined by the decision. For example, probability of particular outcomes, potential reward, or the cost of actions, might all lead to a participant making a response at variance with the output of a contrast comparator. In the framework where sensory input determines perception, perception determines judgments, and this decision in combination with other influences leads to response selection there is room for more than one type of bias. Thus, after perceptual processing you could judge that stimulus A was probably more contrasted than stimulus B, but you might respond B anyway, if for example B was almost always the correct response. This may strike some as a subtle difference, but it can provide a consistent account of the results without the need to invoke a perceptual change in appearance [23]. It is on this basis that Schneider and Komlos [10] argued that equality judgments were the better procedural method, and the decision bias account would explain why participants with expertise for making contrast judgments don’t seem to show the enhancement effect [42].

While both response bias and decision bias are plausible mechanisms for shifting the choice probabilities that ultimately are the basis for claiming that attention changes appearance, neither provides a complete account for the results presented here. Detecting a contrasted stimulus varies with the specifics of the ultimate judgment that the participant made. Neither decision nor response biases easily explain why contrast reports vary between comparison reports and discrimination reports.

Why don’t participants show contrast enhancement when they make direct contrast reports? They are quite capable of judging contrast. There is excellent agreement between the displayed contrast and the judged contrast. Should we conclude that one or the other method is “wrong?” And if so, how do we choose which of the two methods records the “true” effect without being either arbitrary or yielding to our pre-existing theoretical biases? An alternate approach is available. We accept both results, and we abandon our presumption that attentional effects and object appearances are unitary. This conclusion is consistent with all the data. It follows that the stimuli in our task do not have one appearance, but, at least, two. Not only is this conclusion consistent with the data, it also is consistent with the idea that attention is related to importance and prioritization. It just requires that we acknowledge that our situational priorities are not driven only by the sensory data and its variance in salience and learned associations, but also with what it is we want to do or will have to do. Different tasks determine different costs and benefits. When the task is a comparison task it may be that “turning up the volume” (accentuating contrast) may be beneficial, even if it leads to perceptual distortions. On the other hand, when the task is to make a fine discriminating judgment, the costs and benefits may change, and contrast enhancement is no longer observed.

Different behavioral tasks may have different demands. Particular behavioral demands may be better handled by particular physiological mechanisms. When looking for a stimulus against noise, it is best to put greater weight on sensory populations tuned to the stimulus feature [43]. When trying to discriminate between two similar stimuli, it is better to weight sensory populations slightly tuned away from the stimulus feature. To explain a diversity of behavioral effects of attention, one invokes different mixtures of attentional mechanisms. The common observation that cues generally increase neuronal firing, called response gain, could explain increases in perceptual intensity [44]. On the other hand, contrast gain and divisive normalization [45, 46] where neuronal firing patterns change to increase stimulus distinctiveness, explains decreased errors for fine, discriminative judgments without changes in perceived intensity. Noise reduction is another proposed attentional mechanism that also might produce such effects, but, as mentioned earlier, noise reduction should also lead to less false alarms, and this is not what was seen in Experiments 2 and 3. This argument implies some sort of switching mechanism. This is plausible given the behavioral data reported here, and work showing that the different weighting modes optimal for different types of judgments are observed in functional imaging experiments [47, 48].

If one accepts the view that multiple effects of attention might co-exist and depend on the type of experimental protocol, then there is no need to pick the one option among the several active hypotheses regarding attentional mechanisms. Different mechanisms of attention might co-exist. Attention might be multifaceted and there would be room for several of the proposed attentional mechanisms [36, 44, 45, 49–57].

The multiple appearances account is similar in spirit to the the multiple drafts model [58]. According to this multiple drafts model of consciousness, many of the puzzles of conscious experience disappear if one abandons the fixation that there is one place where conscious experience all comes together. Similarly, the multiple appearances account envisions multiple neural and perceptual systems simultaneously operating on overlapping sets of sensory data. With particular laboratory measures we may select distinct sets of these perceptual systems. Thus, what we “see” will depend not only on current sensory evidence, but also on our beliefs, and intentions.
